# Range-Intensity-Profile-Guided Gated Light Ranging and Imaging Based on a Convolutional Neural Network

**DOI:** 10.3390/s24072151

**Published:** 2024-03-27

**Authors:** Chenhao Xia, Xinwei Wang, Liang Sun, Yue Zhang, Bo Song, Yan Zhou

**Affiliations:** 1 Optoelectronic System Laboratory, Institute of Semiconductors, Chinese Academy of Sciences, Beijing 100083, China; xiach406156867@semi.ac.cn (C.X.);; 2 Center of Materials Science and Optoelectronics Engineering, University of Chinese Academy of Sciences, Beijing 100049, China; 3 School of Electronic, Electrical and Communication Engineering, University of Chinese Academy of Sciences, Beijing 100049, China

**Keywords:** range-gated imaging, range-intensity profile, deep learning, depth recovery, light ranging and imaging

## Abstract

Three-dimensional (3D) range-gated imaging can obtain high spatial resolution intensity images as well as pixel-wise depth information. Several algorithms have been developed to recover depth from gated images such as the range-intensity correlation algorithm and deep-learning-based algorithm. The traditional range-intensity correlation algorithm requires specific range-intensity profiles, which are hard to generate, while the existing deep-learning-based algorithm requires large number of real-scene training data. In this work, we propose a method of range-intensity-profile-guided gated light ranging and imaging to recover depth from gated images based on a convolutional neural network. In this method, the range-intensity profile (RIP) of a given gated light ranging and imaging system is obtained to generate synthetic training data from Grand Theft Auto V for our range-intensity ratio and semantic network (RIRS-net). The RIRS-net is mainly trained on synthetic data and fine-tuned with RIP data. The network learns both semantic depth cues and range-intensity depth cues in the synthetic data, and learns accurate range-intensity depth cues in the RIP data. In the evaluation experiments on both a real-scene and synthetic test dataset, our method shows a better result compared to other algorithms.

## 1. Introduction

Depth information provides a powerful means of capturing and presenting high-definition, genuine 3D data. It facilitates accurate quantitative capture of spatial 3D information and reconstruction of an object’s geometric topography. This technological advancement not only overcomes the limitations inherent in traditional 2D imaging but also elevates the level of realism and immersion within a scene. This transformative capability of depth information extends beyond mere image rendering, extending its impact across various fields such as computer vision, medical imaging, robotics, autonomous driving, graphics, virtual reality (VR) and augmented reality (AR) [1,2,3,4,5]. Therefore, depth information unlocks new dimensions of possibility and innovation in numerous applications, and depth sensing has been attracting enduring attention from both academic and industry communities for decades.

Over the years, researchers have been developing various depth-sensing technologies for obtaining high-quality depth information. The mainstream depth-sensing methods can be categorized into passive (i.e., monocular and stereo RGB depth estimation [6,7,8,9]) and active (i.e., scanning light detection and ranging (LiDAR) and time of flight (TOF) camera [10,11]) approaches. For passive approaches, depth estimation from monocular or stereo RGB images offers a cost-effective alternative to obtain depth information. Recently, deep learning has become a research hotspot [12,13,14,15,16], and it has been widely used in image-based depth estimation methods and making great progress to improve depth accuracy [17,18]. These deep-learning-based depth estimation algorithms struggle to achieve depth accuracy comparable to active techniques, and hardly work in low-light environments. Compared to passive approaches, the common active techniques such as scanning LiDAR and TOF cameras integrate an active light source for target illumination, which enables practical and precise depth sensing even in a low-light environment, and can also significantly enhance the depth accuracy, imaging speed and reliability of imaging. However, generally scanning LiDAR instruments are unable to acquire dense point clouds in real time due to their mechanical scanning operation, and TOF cameras suffer from the low spatial resolution of imaging sensors [19].

Different from the scanning LiDAR technique, 3D range-gated imaging eliminates the need for bulky scanning devices and mitigates the constraints imposed by these devices on imaging speed. Different from the TOF camera technique, 3D range-gated imaging utilizes gated cameras with high-spatial resolution, providing more detailed depth maps and high uniformity texture images. Therefore, the 3D range-gated imaging can obtain depth maps with spatial resolution comparable to passive imaging methods at a high frame rate and with a high depth accuracy comparable to LiDAR and TOF cameras [4,20,21,22]. Meanwhile, due to the high gate-shutter speed of gated cameras and the advanced time controlling unit (TCU), range-gated imaging can block out irrelevant background and scattered light and obtain higher quality intensity images than the traditional active depth-sensing methods. In range-gated imaging, gate images with different range-intensity profiles (RIPs) can be formed by the convolution of different laser pulses and camera gate pulses, and the intensities of objects in these images are correlated with their distances. Up to now, different depth recovery algorithms over RIPs have been developed for 3D range-gated imaging. Laurenzis et al. proposed a range-intensity correlation algorithm by at least two gated images with trapezoidal RIPs [22]. Wang et al. developed a range-intensity correlation algorithm based on triangular RIPs to improve the depth resolution [21]. However, the two algorithms assume the RIPs to be a perfect trapezoid or triangle shape. Due to irregularities in laser pulses, camera gate pulses and the effects of light transmission, the real shapes of RIPs are typically irregular, and the two derived range-intensity correlation algorithms are not consistent with real situations, which reduces their precision and accuracy of recovered depth. With the rise of deep-learning methods, Gruber et al. used a multilayer perceptron to obtain depth information from three gated images with free-shaped RIPs [23]. Gruber et al. generalized a Gated2Depth algorithm by utilizing convolutional neural network (CNN) and generative adversarial network (GAN) to learn depth information from three gated images with free-shaped RIPs [4]. However, these deep-learning methods need a large real-scene dataset. In the previous multilayer perceptron algorithm, they captured gated images and depth maps by placing diffuse targets with different reflectance from 10 m to 100 m and collected 10,000 samples. In the Gated2Depth algorithm, a gated dataset has been collected by a gated camera and LiDAR over 4000 km driving. Although commercial gated cameras have become available, it requires significant time and monetary costs to collect enough training data.

From passive depth-sensing methods to active depth-sensing methods, we believe that light ranging and imaging (LiRAI) will be a hot topic of depth sensing, since the acquisition of dense point cloud with range information and high-resolution intensity images at the same time are important for academic and industry applications. In fact, the 3D range-gated imaging is a LiRAI technique. To overcome the disadvantages of the above algorithms for 3D range-gated imaging, we propose a method of range-intensity-profile-guided gated light ranging and imaging based on a convolutional neural network. The method is named as RIP-Gated3D. Specifically, we propose the following contributions:

(1) The method RIP-Gated3D is proposed to obtain depth maps of high spatial resolution and high accuracy using the gated LiRAI system;
(2) A network which utilize both “range-intensity” depth cues and semantic depth cues in two gated images is proposed to generate depth maps;
(3) We generate synthetic training data using the real RIP of our gated LiRAI system and data from GTAV, and our network is mainly trained on synthetic training data and finetuned with small number of real range-intensity profile data;
(4) We validate our method on a synthetic dataset and real-scene dataset, the network generates depth maps of high accuracy and solves the problems of distorted and blurry edges shown in other deep-learning based methods.

The remainder of this paper is organized as follows. Section 2 describes the principle of the RIP-Gated3D method, including the specifics of our gated LiRAI system, the structure of range-intensity ratio and semantic network (RIRS-net), the synthetic dataset generation method and the training details. Section 3 shows the experimental results of the proposed method. Finally, Section 4 draws the conclusions.

## 2. RIP-Gated3D Method

### 2.1. General Technical Solution

**Figure 1:**
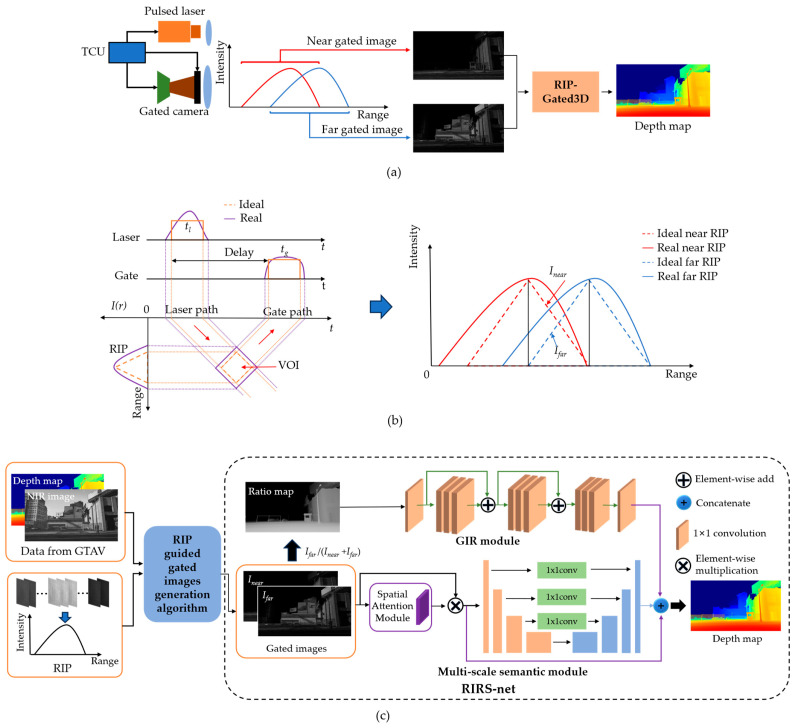
The principle of RIP-Gated3D method: (**a**) General technical solution; (**b**) Ideal RIPs and real RIPs of two gated images with $t_{l}=t_{g}$; (**c**) Our RIP-Gated3D method.

Figure 1 shows the principle of the proposed RIP-Gated3D method. In Figure 1a, a typical gated LiRAI system consists of a pulsed laser as the illumination source, a gated camera and a time controlling unit (TCU). When the system works, the pulsed laser emits a laser pulse $p\left(t\right)$ with a pulse width of $t_{l}$ at time t=0, illuminating the scene. After a certain delay time of τ, the gated camera opens for a given gate time of $t_{g}$, during which the reflected light from volumes of interest (VOI) are recorded as illustrated in the left of Figure 1b. Assuming a target is located at the distance of r, the target reflected light signal $I_{i}\left(r\right)$ received by the gated camera can be depicted as:

(1) $$I_{i}\left(r\right)=\kappa \alpha C_{i}\left(r\right)=\kappa \alpha \int_{0}^{\infty }p\left(t-\frac{2r}{c}\right)g\left(t-\tau \right)dt$$

where α represents the reflectance of the diffuse surface, $\kappa =\frac{1}{r^{2}}$, $C_{i}\left(r\right)$ is the RIP of the VOI formed under the convolution of laser pulse $p\left(t\right)$ and gate pulse $g\left(t\right)$, and c is the speed of light. Equation (1) is applicable in atmospheric environments because the attenuation of lasers due to the atmosphere can be neglected within the gated LiRAI system’s operating range.

A typical range-intensity correlation algorithm utilizes two gated images to recover depth, and the shapes of the laser pulse $p\left(t\right)$ and gate pulse $g\left(t\right)$ should be rectangular. For the triangular range-intensity correlation algorithm with a higher range precision than the trapezoidal algorithm with $t_{g}=t_{l}$ [21], the ideal RIPs of two gated images are depicted by dashed lines in Figure 1b. Then, in their overlapping volume, the target range or depth can be calculated by Equation (2):

(2) $$r=\frac{c}{2}\left(\tau +\frac{I_{\mathrm{far}}}{I_{\mathrm{near}}+I_{\mathrm{far}}}t_{l}\right)$$

where r is the target range from the gated LiRAI system, c is the speed of light, τ represents the time delay of the near gated image and $t_{l}$ represents the laser pulse width. $I_{\mathrm{near}}$ denotes the intensity in the near gated image and $I_{\mathrm{far}}$ denotes the intensity in the far gated image. Equation (2) works well when the shapes of the laser pulse and gate are both strictly rectangles. However, the real RIPs are usually not rectangles. Figure 1b shows the real shape of RIPs by solid lines. As a result, calculating depth by the traditional range-intensity correlation methods suffers a decrease in accuracy.

Therefore, a RIP-Gated3D method is proposed to recover high-accuracy depth information from gated images in Figure 1c. In the method, synthetic gated images are generated by a RIP-guided gated image generation algorithm, and a RIRS-net is designed to recover depth from gated images. The RIRS-net is mainly trained on a large dataset of synthetic gated images with a ground truth depth map. To obtain the real RIP for RIRS-net, we place a white screen at a fixed distance and collect a sequence of gated images by changing the time delay of our gated LiRAI system. The real RIP is obtained through mathematical fitting with this sequence of RIP data. Additionally, synthetic depth maps and near infrared (NIR) images are extracted from Grand Theft Auto V (GTAV) [24]. Then the synthetic gated images are generated by applying the measured RIP on NIR images and corresponding depth maps of GTAV synthetic scenes. The RIRS-net contains a gated image ratio (GIR) module and multi-scale semantic module. The GIR module consists of 4 residual blocks. Meanwhile, the GIR module only uses convolutional layers with 1×1 kernels, utilizing the “range-intensity” depth cues to help to recover the edge errors in the multi-scale semantic module. The multi-scale semantic module consists of U-net architecture, spatial attention module and 1×1 kernels before skip connections, and this module utilizes downsample operations and 3×3 kernels to learn from semantic depth cues. We train our network with 1250 pairs of synthetic gated images and use the RIP data to fine-tune it. The RIRS-net is able to exploit both range-intensity depth cues and semantic depth cues while training on the synthetic data and exploit accurate range-intensity depth cues while training on the RIP data. Finally, the depth maps with high accuracy are reconstructed.

### 2.2. Dataset

#### 2.2.1. Real Range-Intensity Profile

**Figure 2:**
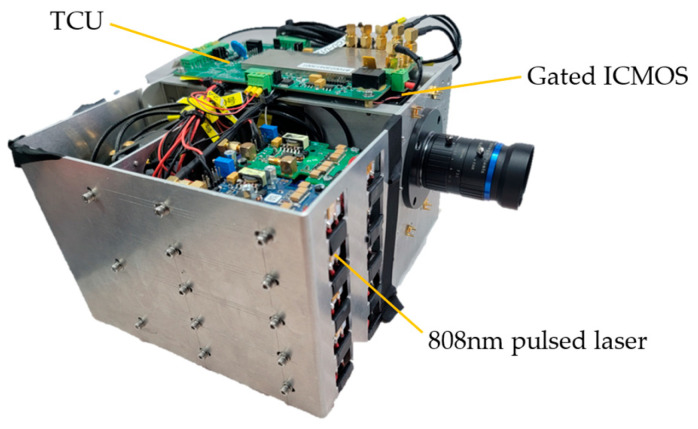
The gated LiRAI system.

Our method relies on the real RIP of a given gated LiRAI system to generate synthetic gated images as the training data. In our gated LIRAI system as shown in Figure 2, a pulsed laser of 808 nm with a typical pulse repetition frequency of 30 kHz is used for illumination, and a gated intensified complementary metal oxide semiconductor (ICMOS) with 1604 × 1108 pixels is used as a gated camera to capture 8-bit gated images. The TCU implemented by the field programmable gate array (FPGA) provides the desired time sequence for the pulsed laser and the gated ICMOS.

**Figure 3:**
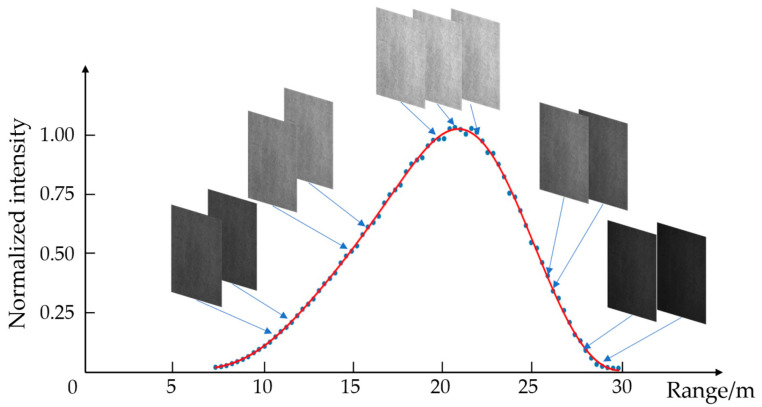
The process of acquiring a sequence of gated images and fitting the RIP. The dots represent the gray-scale values of the gated images, and the red line represents the fitted RIP.

In the experiments, the gate width, laser pulse width and delay between the two gated images are set to 50 ns. To obtain the real RIP, a white screen is placed at a distance of 20 m. One changes the time delay of the gated LiRAI system with a step of 2 ns to obtain a sequence of gated images. The start delay is set to 48 ns, and the end delay is set to 198 ns. We collect 76 gated images in total to obtain the RIP. For each gated image in this sequence, the mean gray-scale value of the center area in the gated image is calculated, and then these gray-scale values are normalized. The RIP range is from 7.2 m to 29.7 m according to the time delay from 48 ns to 198 ns. Through mathematical fitting by using the Gaussian mixture model, the real RIP is plotted in a red solid line in Figure 3.

#### 2.2.2. Real Data

In the process of fitting the real RIP of our gated LiRAI system, we capture a sequence of gated images of the white screen and record their corresponding depth information. These RIP data are also used to fine-tune RIRS-net, and the details are shown in Section 2.4. For the real test dataset, two diffuse targets with the reflectance of 10% and 90% are placed at distances of approximately 12, 14, 16, 18 and 20 m away from the gated LiRAI system. At each distance, we change the time delay of the gated LiRAI system with a step of 1 ns and set the delay between the near gated image and the far gated image to 50 ns to collect 30 pairs of gated images. The corresponding depth maps are captured with a LiDAR system. These gated images and depth maps compose the real test dataset.

#### 2.2.3. Synthetic Data

**Figure 4:**
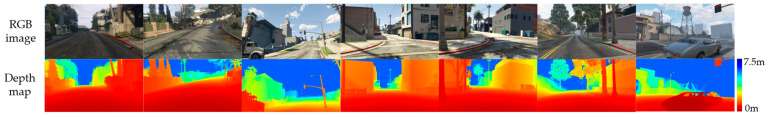
Examples of raw data from GTAV. Each scene contains an RGB image and corresponding depth map.

To obtain a synthetic dataset, we collect large-scale synthetic data from the game GTAV and renderdoc is used to read out the drawcall while playing the game [25,26]. RGB images and depth maps contained in the drawcall data are of interest to us, and data examples are shown in Figure 4. Since the gated LiRAI system works at the wavelength of 808 nm, the RGB images are transformed into NIR images to obtain image characteristics similar to those of the gated LiRAI. We shift the real RIP to obtain a near RIP $C_{\mathrm{near}}\left(r\right)$ and a far RIP $C_{\mathrm{far}}\left(r\right)$ with the Equations (3) and (4) below:

(3) $$C_{\mathrm{near}}\left(r\right)=C\left(r+r_{\mathrm{near}}\right)$$

(4) $$C_{\mathrm{far}}\left(r\right)=C\left(r+r_{\mathrm{far}}\right)$$

where $r_{\mathrm{near}}$ denotes the shifted distance between the near RIP and the real RIP, and $r_{\mathrm{far}}$ denotes the shifted distance between the far RIP and the real RIP.

The synthetic gated images are obtained with Equation (1). In the near gated image, the near RIP $C_{\mathrm{near}}\left(r\right)$ represents the ratio of intensity in the near gated image to the NIR image, and in the far gated image, the far RIP $C_{\mathrm{far}}\left(r\right)$ represents the ratio of intensity in the far gated image to the NIR image. The NIR images of $I_{\mathrm{NIR}}$, depth maps of d, the near RIP $C_{\mathrm{near}}\left(r\right)$ and the far RIP $C_{\mathrm{far}}\left(r\right)$ together generate near gated images $I_{\mathrm{near}}$ and far gated images $I_{\mathrm{far}}$ by Equations (5) and (6):

(5) $$I_{\mathrm{near}}\left(x,y\right)=C_{\mathrm{near}}\left(d\left(x,y\right)\right)\times I_{\mathrm{NIR}}\left(x,y\right)$$

(6) $$I_{\mathrm{far}}\left(x,y\right)=C_{\mathrm{far}}\left(d\left(x,y\right)\right)\times I_{\mathrm{NIR}}\left(x,y\right)$$

where $\left(x,y\right)$ represents the column and row of the pixel in the image.

**Figure 5:**
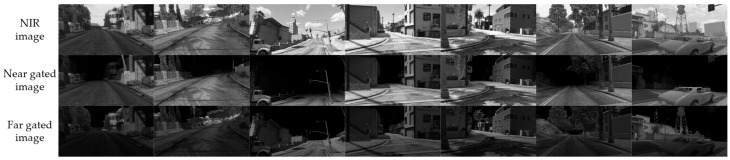
Examples of synthetic data. Each scene contains a simulated NIR image, a near gated image and a far gated image.

We simulate 1436 sample scenes, including 1250 for training and 186 for testing. Examples of synthetic data are shown in Figure 5.

### 2.3. Network Architecture

The RIRS-net consists of two parts: a GIR module and a multi-scale semantic module. The input of the GIR module is a ratio map which is obtained using the far gated image divided by the sum of the near and far gated images. The input of multi-scale semantic module are two gated images. The output feature maps of the two modules are concatenated to obtain the final depth map. The GIR module exploits the range-intensity depth cues contained in the gray-scale values of the two gated images, and the multi-scale semantic module helps to exploit the semantics in the scene. The details of the GIR module and multi-scale semantic module are described as follows.

#### 2.3.1. GIR Module

Different from traditional monocular or binocular depth recover networks which rely completely on the semantic information in the scene, the gated images contain depth cues generated from RIPs. These depth cues allow us to exploit the relationship between the corresponding pixels $I_{\mathrm{near}}\left(x,y\right)$ and $I_{\mathrm{far}}\left(x,y\right)$ in the two gated images $I_{\mathrm{near}}$ and $I_{\mathrm{far}}$. We design the GIR module consisting of convolutional layers with 1×1 kernels only. This module is composed of 4 residual blocks, and each block consists of 3 convolutional layers with 1×1 kernels. No pooling operation is applied, and convolutional layers with a stride of 1 are applied. ReLU [27] activation is followed by the convolutional layer. Residual blocks are widely applied in convolutional neural networks since the deep residual network was proposed by Kaiming He [14]. Shortcuts in the residual network allow the model to learn residual functions and help in training a deep network. The GIR module pixel-independently estimates depth from two gated images, without the semantic information between context pixels. Without convolutional kernels larger than 1×1 and any downsample operation, for all the pixels in the output of GIR module, they are not influenced by the pixels nearby, which helps to recover the distorted and blurry edges in the output of the multi-scale semantic module.

#### 2.3.2. Multi-Scale Semantic Module

The GIR module alone can work to make depth estimation from gated images, and the accuracy of estimated depth increases when the network becomes deeper. However, no pooling layers and only using 1×1 convolutional kernels fails to exploit the semantics in the gated images. In addition, without downsample and upsample operations, the GIR module still struggles to generate high-accuracy depth maps even with a deeper network architecture. Therefore, we propose the multi-scale semantic module which is composed of a U-net structure, spatial attention module and 1×1 kernels before skip connections. The U-net structure, which gives higher-level neurons large receptive fields, captures more global semantic information [28]. The contracting path of U-net captures context in images and the symmetric expanding path enables the output feature map to maintain the same size as the input, so that U-net is suitable for pixel-wise tasks. The multi-scale semantic module consists of 4 pairs of convolutions with a max pooling operation after each pair. The encoder of U-net produces internal feature maps 1/2, 1/4, 1/8 and 1/16 of the original input size. The decoder consists of 8 convolutional layers and 4 bilinear operations to upsample the contracted feature map.

To reduce the loss of information during downsample and upsample operations, skip connections are applied in the original U-net architecture. The contracting path and expanding path feature maps of the same size are concatenated for future convolution and upsampling. In addition to this, we add convolutional layers with 1×1 kernels before skip connections. Layers of 1×1 kernels with a stride of 1 maintain the resolution of the feature maps from the encoder, and help the encoder feature maps to exploit the depth cues between channels while preserving edge information. As a result, the multi-scale semantic module obtains semantic information from gated images, and simultaneously reduces the loss of edge information.

**Figure 6:**
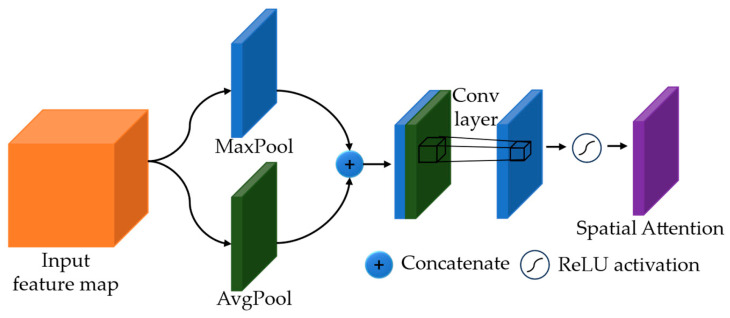
Architecture of spatial attention module.

Inspired by traditional range-intensity correlation algorithms [21,22], they estimate depth values in the overlapping volume of the two gated images and discard other regions. Since a non-overlapping area has a lack of depth cues, we design a spatial attention module which dynamically allocates attention to different parts of the image, allowing the model to prioritize important regions while suppressing irrelevant or less important ones [2,3]. This module enables our network to give more emphasis to the overlapping regions while downplaying others. As shown in Figure 6, to compute the spatial attention, we apply average-pooling and max-pooling operations to the input feature map along the channel axis and generate two feature maps. Then, the average-pooled feature map and the max-pooled feature map are concatenated and convolved by a convolution layer with 3×3 kernels to generate the output spatial attention.

### 2.4. Implementation Details

We use data augmentation to increase the number of training samples. The input gated images, ratio maps and corresponding ground truth are transformed by using gray-scale transformations and horizontal/vertical flips with a 0.5 chance, and the gated images are normalized. The augmented images are downsampled to the chosen input size of 480×256.

We implement the RIRS-net with Pytorch 1.13.1 [29], and train on a single NVIDIA GeForce RTX 3090 with 24 GB of GPU memory. The input size of our network is 480×256. The computation complexity of RIRS-net is 90.19 GFLOPs. The loss function is L2 loss:

(7) $$\mathrm{loss}=\frac{1}{n}\overset{W}{\underset{i}{\sum }}\overset{H}{\underset{j}{\sum }}\delta_{ij}{\left(d_{ij}^{\mathrm{recovered}}-d_{ij}^{\mathrm{truth}}\right)}^{2}$$

where *n* is the number effectives pixels in the depth map, i and j represent the rows and columns, $\delta_{ij}$ equals 1 when the ground truth of the pixel is within the range of 0–7.5 m and otherwise equals 0 since the range of overlapping volume of the near gated image and far gated image is 7.5 m, $d_{ij}^{\mathrm{recovered}}$ is the estimated depth information and $d_{ij}^{\mathrm{truth}}$ is the ground truth.

The Adam optimizer [30] with $\beta_{1}=0.9$ and $\beta_{2}=0.999$ is applied to train the RIRS-net. In total, the network is trained for 60 epochs with the synthetic training data and 10 epochs with the RIP data. The initial learning rate is set as 0.00005, and for every 7 epochs the learning rate is halved.

## 3. Experiment and Results

### 3.1. Experiment

We evaluate our RIP-Gated3D method by comparing it against other range-intensity depth estimation methods including the numerical triangular method [21], multilayer perceptron [23] and Gated2Depth network [4]. Two classic monocular depth estimation networks including FCRN [31] and DORN [32] are also used for comparative experiments. Multilayer perceptron, Gated2Depth network, FCRN and DORN are trained on our synthetic and RIP dataset. It should be noted that the original input of the multilayer perceptron and Gated2Depth network are both three gated images. In the section, we evaluate all the methods with the input of two gated images by the metrics mean square error (RMSE), mean absolute error (MAE), absolute relative error (AbsRel) and $\delta_{k}<1.25^{k}$ for k∈1, 2, 3 [4,5,15,16,33]. MAE describes the average absolute difference between recovered depth and ground truth, providing a straightforward indication of how far the predictions are from the actual values on average. MAE is defined in Equation (8). RMSE describes the square root of the average squared error, and it gives more weight to large errors compared to MAE because of the squaring operation. RMSE is defined in Equation (9). Abs Rel describes the absolute difference scaled by the reciprocal of the ground truth, and it is particularly useful for comparing errors across different scales of data. AbsRel is calculated according to Equation (10). In Equation (11), $\delta_{k}$ describes the percentage of recovered depth d within $1.25^{k}$ relative to the ground truth.

(8) $$\mathrm{MAE}=\frac{1}{n}\overset{W}{\underset{i}{\sum }}\overset{H}{\underset{j}{\sum }}\delta_{ij}\left|d_{ij}^{\mathrm{recovered}}-d_{ij}^{\mathrm{truth}}\right|$$

(9) $$\mathrm{RMSE}=\sqrt{\frac{1}{n}\overset{W}{\underset{i}{\sum }}\overset{H}{\underset{j}{\sum }}\delta_{ij}{\left(d_{ij}^{\mathrm{recovered}}-d_{ij}^{\mathrm{truth}}\right)}^{2}}$$

(10) $$\mathrm{AbsRel}=\frac{1}{n}\overset{W}{\underset{i}{\sum }}\overset{H}{\underset{j}{\sum }}\delta_{ij}\frac{\left|d_{ij}^{\mathrm{recovered}}-d_{ij}^{\mathrm{truth}}\right|}{d_{ij}^{\mathrm{truth}}}$$

(11) $$\delta_{k}<1.25^{k}=\frac{1}{n}\overset{W}{\underset{i}{\sum }}\overset{H}{\underset{j}{\sum }}\delta_{\left\{\max\left(\frac{d_{ij}^{\mathrm{recoverd}}}{d_{ij}^{\mathrm{truth}}},\;\frac{d_{ij}^{\mathrm{truth}}}{d_{ij}^{\mathrm{recovered}}}\right)<1.25^{k}\right\}}$$

### 3.2. Results on Synthetic Dataset

**Figure 7:**
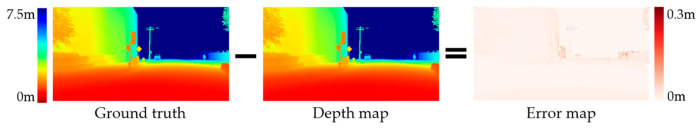
The process of generating an absolute error map.

Firstly, these methods are evaluated on the synthetic test dataset from Section 2.2.3. We calculate the difference between predicted depth and ground truth in every pixel. The absolute error maps are used to visualize the difference between the recovered depth maps and ground truth. An example of generating an error map is shown in Figure 7.

**Figure 8:**
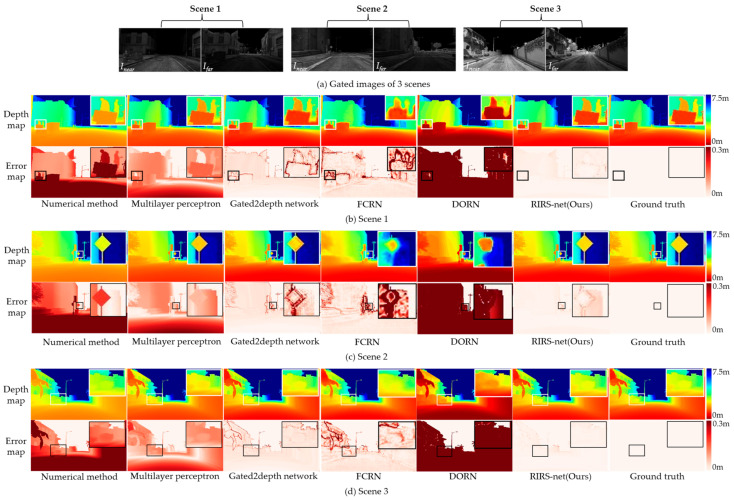
Results for our method and other methods on synthetic test data. Our method generates accurate depth maps over all the ranges: (**a**) Gated images of 3 scenes; (**b**) Scene 1; (**c**) Scene 2; (**d**) Scene 3.

Figure 8 visualizes the depth maps recovered by different methods and corresponding ground truth in three synthetic scenes. Meanwhile, we provide absolute error maps below each depth map. White boxes are used to magnify the regions with more details in the depth maps and black boxes are used to magnify the corresponding regions in the error maps, making the comparison more intuitive. It is obvious that the numerical method and DORN are struggling to recover accurate depth. The multilayer perceptron performs better than the above two methods but still obtains depth maps of a low accuracy. The Gated2Depth network and FCRN recover depth accurately in most regions but make mistakes in the edge regions. Our method recovers depth which closely aligns with ground truth in the vast majority of areas. The depth maps exhibit clear differentiation between different objects, with distinct outlines and noticeable details. Compared to the Gated2Depth method, the accuracy of depth in the edge regions shows a significant improvement in our method. The detailed results are shown in Table 1. After approximation, the $\delta_{1}$, $\delta_{2}$ and $\delta_{3}$ are one in our method. The MAE of the recovered depth map is 0.014 m, with an error only about one-third of the Gated2Depth method, the RMSE is only one-fourth of the Gated2Depth method and the AbsRel is half of the Gated2Depth method. The results show our method outperforms all of the other reference methods and recovers depth maps of higher accuracy.

### 3.3. Results on Real-Scene Dataset

**Figure 9:**
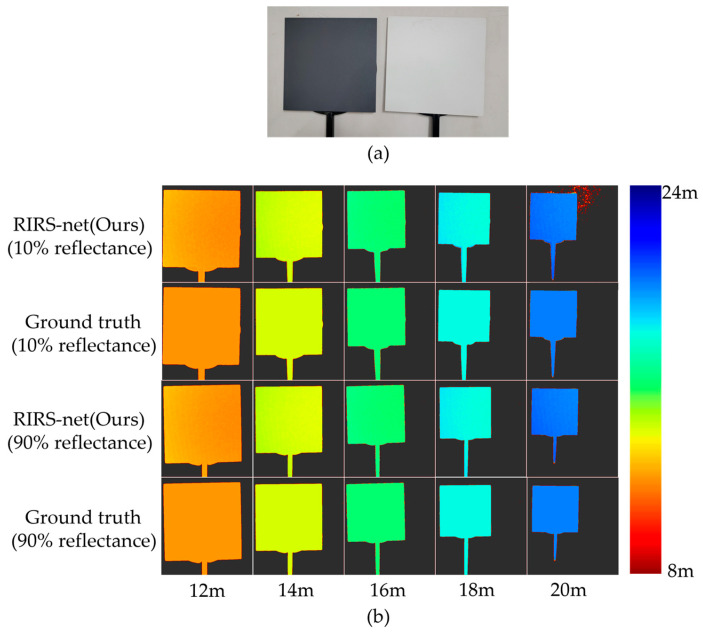
Results on the real-scene test data: (**a**) Diffuse targets. Left: reflectance of 10%. Right: reflectance of 90%; (**b**) Depth maps of targets placed from 12 m to 20 m.

We compare the results obtained by our method to other methods on a real-scene test dataset from Section 2.2.2. Figure 9 demonstrates the ground truth and depth maps estimated with the RIP-Gated3D method.

We calculate the average depth of all points on the diffuse targets and compare it with the ground truth. The detailed results are shown in Table 2. For diffuse targets with different reflectance, our method recovers depth from gated images with an MAE less than 5 cm and an absolute relative error less than 0.4% and outperforms the other methods on the real-scene dataset. Compared to the second best method of Gated2Depth in Table 2, our method reduces the MAE by 20.3%, reduces the RMSE by 8.7% and reduces the AbsRel by 8.7%.

To verify the effectiveness of the two modules in RIRS-net, we conduct ablation studies on the real test data. We train the GIR module and multi-scale semantic module respectively and test them. The results in Table 2 indicate that a single module recovers depth with lower accuracy compared to our RIRS-net.

**Figure 10:**
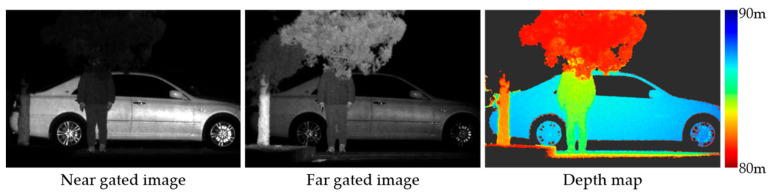
Real-world recovered depth map based on the RIP-Gated3D method.

Gated images in real-world conditions are captured to make qualitative analysis for our RIP-Gated3D method. The near gated image, far gated image and corresponding depth map recovered using the RIP-Gated3D method are shown in Figure 10. In the depth map, the edges of the person, the tree, and the vehicle are very clear, which indicates that our method has potential applications in fields such as autonomous driving and robotics.

### 3.4. Ablation Study

We conduct ablation studies to compare and analyze the performance of the proposed GIR module, multi-scale semantic module and spatial attention module in the multi-scale semantic module.

Firstly, we analyze the effectiveness of the GIR module and multi-scale semantic module. We train the GIR module and multi-scale semantic module alone. Shown in Table 3 and Table 4, the results indicate that a single module is unable to generate depth maps of high accuracy. Our proposed RIRS-net combines the two modules, promoting the performance on both synthetic test data and real-scene test data.

Secondly, we analyze the effect of the spatial attention module in the multi-scale semantic module. Compared to a multi-scale semantic module without a spatial attention module, the performance of the multi-scale semantic module is slightly better. Our RIRS-net also performs better than RIRS-net without a spatial attention module on both synthetic test data and real-scene test data.

In conclusion, the results on both test datasets demonstrate the effectiveness of GIR module, multi-scale semantic module and spatial attention module used in our RIRS-net.

## 4. Conclusions and Discussion

In this work, we propose the method of RIP-Gated3D to recover depth from gated images. It can realize LiRAI including intensity images and depth maps with high spatial resolution and high accuracy. In the proposed method, a real RIP is obtained through mathematical fitting from RIP data captured with a gated LiRAI system. Synthetic intensity images and corresponding depth maps are captured from GTAV. A RIP-guided gated image generation algorithm is designed to generate synthetic training data for the RIRS-net using data from GTAV and the real RIP. We train our network with synthetic training data and fine-tune it with RIP data. The proposed RIRS-net learns both semantic depth cues and range-intensity depth cues contained in the synthetic data and learns accurate range-intensity depth cues contained in the RIP data. Compared to other methods, we train the RIRS-net with a small number of real gated images and our network outperforms other classic depth estimation methods of 3D range-gated imaging.

Our gated LiRAI system typically works at 2–30 Hz. However, recovering the depth of a high-speed object from gated images is challenging, which needs to be studied in future work. An interesting direction for the future work is to estimate depth from a single gated image, which helps to improve the imaging speed and has advantages in imaging moving objects.

## Figures and Tables

**Table 1:** Comparison of our network, the numerical method, multilayer perceptron, Gated2Depth network, FCRN and DORN on the synthetic dataset. The optimal performance is highlighted using bold font.

Method	MAE [m]	RMSE [m]	AbsRel [%]	$\delta_{1}$	$\delta_{2}$	$\delta_{3}$
Numerical method	0.624	0.686	71.14	0.367	0.523	0.678
Multilayer perceptron	0.249	0.288	30.20	0.589	0.780	0.936
Gated2Depth network	0.040	0.088	2.00	0.996	0.999	**1.000**
FCRN	0.081	0.179	4.48	0.988	0.997	0.999
DORN	0.795	0.825	63.70	0.155	0.280	0.363
RIRS-net (Ours)	**0.014**	**0.022**	**0.91**	**1.000**	**1.000**	**1.000**

**Table 2:** Comparison of our network, the numerical method, multilayer perceptron, Gated2Depth network, FCRN and DORN on real-scene dataset. The optimal performance is highlighted using bold font.

Method	Target Reflectance	MAE [m]	RMSE [m]	AbsRel [%]
Numerical method	10%	0.554	0.651	3.57
	90%	0.563	0.656	3.62
Multilayer perceptron	10%	0.060	0.169	0.93
	90%	0.044	0.159	0.87
Gated2Depth network	10%	0.052	0.073	0.39
	90%	0.037	0.059	0.31
FCRN	10%	0.053	0.075	0.40
	90%	0.034	0.063	0.33
DORN	10%	0.808	0.814	5.24
	90%	0.812	0.817	5.26
RIRS-net (Ours)	10%	**0.045**	**0.067**	**0.36**
	90%	**0.027**	**0.054**	**0.28**

**Table 3:** Ablation of RIRS-net on synthetic dataset. The optimal performance is highlighted using bold font.

Method	MAE [m]	RMSE [m]	AsRel [%]	$\delta_{1}$	$\delta_{2}$	$\delta_{3}$
GIR module	0.063	0.087	4.24	0.996	**1.000**	**1.000**
Multi-scale semantic module without spatial attention module	0.037	0.073	2.10	0.997	**1.000**	**1.000**
Multi-scale semantic module	0.028	0.050	1.88	0.999	**1.000**	**1.000**
RIRS-net without spatial attention module	**0.014**	0.023	1.24	**1.000**	**1.000**	**1.000**
RIRS-net (Ours)	**0.014**	**0.022**	**0.91**	**1.000**	**1.000**	**1.000**

**Table 4:** Ablation of RIRS-net on the real-scene dataset. The optimal performance is highlighted using bold font.

Method	Target Reflectance	MAE [m]	RMSE [m]	AbsRel [%]
GIR module	10%	0.064	0.108	0.57
	90%	0.039	0.073	0.39
Multi-scale semantic module without spatial attention module	10%	0.050	0.072	0.39
	90%	0.036	0.060	0.32
Multi-scale semantic module	10%	0.049	0.071	0.38
	90%	0.033	0.060	0.32
RIRS-net without spatial attention module	10%	0.046	**0.067**	**0.36**
	90%	0.028	**0.054**	**0.28**
RIRS-net (Ours)	10%	**0.045**	**0.067**	**0.36**
	90%	**0.027**	**0.054**	**0.28**

## Data Availability

Data underlying the results presented in this paper are not publicly available at this time but may be obtained from the authors upon reasonable request.
